# A reflection on 30 years of the Family Health Strategy

**DOI:** 10.1590/0102-311XEN006226

**Published:** 2026-06-22

**Authors:** James Macinko

**Affiliations:** 1 University of California, Los Angeles, U.S.A.

A lot can happen in 30 years. In the case of health policy, 30 years is nearly long enough for ideas that were once unpopular to reemerge as popular. While we might like to think these trends in health policy are due to the accumulation of knowledge and best practices, often they are instead driven by changing political winds and priorities ^1^. By its very nature, our field is situated within the public realm, and given how closely public health topics touch people’s lives, it is to be expected that our work is particularly subject to these winds of change.

For these reasons, I find it all the more remarkable that a pilot project conducted in a poor region of Brazil was identified as successful. Even given early international recognition of its success ^2^, I think few people would have imagined that this project, out of thousands of health care-related interventions conducted worldwide in very different settings, would become the new global flagship, championing a new vision of what primary health care could accomplish. That the program would evolve into the coherent Family Health Strategy (FHS), be successfully replicated across Brazil’s thousands of diverse municipalities, provide a blueprint for how to scale up community-based health interventions, or that it would contribute to real improvements in health care access, health outcomes, and health equity in a country whose health system had previously underperformed seems almost miraculous.

But of course, the story of the FHS is not one of mere chance. It is a story of human agency and a testament to both ingenuity and perseverance. While it is difficult to pinpoint any specific action that explains FHS’s resilience, I think it should be clear that the work of developing, refining, expanding, and improving the strategy has been, and will continue to be, the life project of countless individuals. The tireless dedication and resilience of health workers, local and national staff, administrators at every government sphere, research teams from across Brazil and the world, advocates, and political leaders of many different backgrounds have sustained the FHS and continue to maintain it in the face of new and emerging threats.

Writing from the United States, one lesson strikes me as profoundly important: while we can design interventions that meet all relevant technical criteria, perform evaluations and quality improvement initiatives to the highest scientific standards, and develop flawlessly detailed implementation plans, without the sustained dedication of a cadre of truly visionary leaders and activists dedicated to the achievement of a collective goal, these initiatives often fail. For this reason, the article *Thirty Years of the Family Health Strategy in the Brazilian Unified National Health System: Milestones, Advances, Setbacks and Challenges* ^3^ is particularly salient. It highlights distinct inflection points in the history of the development of the FHS and reflects on active and essential debates currently unfolding within the world’s largest universal community-based primary health care initiative.

While many aspects of the FHS’s evolution and impact have been well documented, one issue addressed in the essay struck me as particularly impressive: despite mounting pressure - whether financial, political, or operational - the vision of the FHS has remained deeply rooted in the humanitarian and egalitarian spirit of *Alma Ata*. The later integration of oral health teams, recognition of the need for greater multidisciplinarity through the Family Health Expanded Centers (NASF, acronym in Portuguese), renewed emphasis on quality improvement, and innovations developed through the More Doctors Program, among many other innovations, all point to an evolving and incremental vision of what primary health care can and should be in order to address today’s complex health challenges.

Clearly, not all of these initiatives have been equally successful. Some actions - such as the 2017 changes to the Brazilian National Primary Health Care Policy (PNAB, acronym in Portuguese) that led to the development of more limited, part-time primary care teams; the reductive conception of “community” advanced by the Previne Brasil program; and the continued underfinancing of the Brazilian Unified National Health System (SUS, acronym in Portuguese) - could each, on their own, have spelled the end of the FHS ^4^.

The article ^3^ also reminds us of the many challenges ahead. These include persistent underfinancing; the availability and distribution of human resources; the achievement of comprehensive and integrated care; structural inequalities that continue to drive stark health inequities; and ongoing uncertainty regarding the public nature of the SUS. Some of these issues are unique to Brazil, but most are not.

Despite these challenges, it is this collective and comprehensive vision that has, in my opinion, sustained the FHS through radically different political regimes, major financial crises, and even a global pandemic. On this 30th anniversary, let us take a moment to look back with pride and gratitude before resuming the arduous work of realizing the ambitious and valuable vision of a strong and resilient primary health care model - one that continues to inspire people around the world and that every day comes closer to fulfilling its great promise.

## Data Availability

Not applicable.
