# Supplementary material for: A reflection on 30 years of the Family Health Strategy
Source: Cad Saude Publica. 2026 Jun 22;42:e00006226. doi: 10.1590/0102-311XEN006226 (PMC13286398; doi:10.1590/0102-311XEN006226)
Supplement: Translation

# Translation: Uma reflexão sobre 30 anos da Estratégia Saúde da Família

**DOI:** 10.1590/0102-311XPT006226

**Authors:** James Macinko

**Affiliations:** 1 University of California, Los Angeles, U.S.A.

Muita coisa pode acontecer em 30 anos. No campo das políticas de saúde, 30 anos é tempo suficiente para que ideias antes impopulares ressurjam como populares. Embora possamos querer acreditar que essas tendências nas políticas de saúde resultem do acúmulo de conhecimento e de boas práticas, elas frequentemente são impulsionadas por mudanças nos ventos e prioridades políticas ^1^. Por sua própria natureza, nosso campo situa-se no âmbito público e, dado o quanto os temas de saúde pública afetam a vida das pessoas, é esperado que nosso trabalho esteja particularmente sujeito a essas mudanças.

Por essas razões, considero ainda mais notável que um projeto-piloto conduzido em uma região pobre do Brasil tenha sido reconhecido como bem-sucedido. Mesmo com o reconhecimento internacional precoce de seu êxito ^2^, acredito que poucas pessoas teriam imaginado que esse projeto, dentre milhares de intervenções relacionadas à saúde realizadas em todo o mundo em contextos muito diferentes, se tornaria o novo modelo de referência global, defendendo uma nova visão do que a atenção primária à saúde poderia alcançar. Que o programa viesse a se consolidar como a coerente Estratégia Saúde da Família (ESF), fosse replicado com sucesso nos milhares de municípios brasileiros, servisse de modelo para a expansão de intervenções comunitárias de saúde, ou que contribuísse para melhorias concretas no acesso à saúde, nos desfechos clínicos e na equidade em saúde em um país cujo sistema de saúde havia apresentado desempenho abaixo do esperado parece quase miraculoso.

Mas, evidentemente, a história da ESF não é uma história de mero acaso. É uma história de agência humana e um testemunho tanto de engenhosidade quanto de perseverança. Embora seja difícil identificar uma ação específica que explique a resiliência da ESF, creio que deve ficar claro que o trabalho de desenvolver, aprimorar, expandir e melhorar a estratégia tem sido, e continuará sendo, o projeto de vida de inúmeros indivíduos. A incansável dedicação e resiliência de profissionais de saúde, equipes locais e nacionais, gestores em todas as esferas governamentais, grupos de pesquisa de todo o Brasil e do mundo, defensores da causa e líderes políticos dos mais variados perfis sustentaram a ESF e continuam a mantê-la diante de ameaças novas e emergentes.

Escrevendo dos Estados Unidos, uma lição me parece profundamente importante: embora possamos elaborar intervenções que atendam a todos os critérios técnicos pertinentes, realizar avaliações e iniciativas de melhoria da qualidade segundo os mais elevados padrões científicos e desenvolver planos de implementação impecavelmente detalhados, sem a dedicação contínua de um conjunto de líderes verdadeiramente visionários e ativistas dedicados à realização de um objetivo coletivo, essas iniciativas frequentemente fracassam. Por essa razão, o artigo *30 Anos da Estratégia Saúde da Família no Sistema Único de Saúde: Marcos, Avanços, Retrocessos e Desafios* ^3^ é particularmente relevante. Ele destaca pontos de inflexão distintos na história do desenvolvimento da ESF e reflete sobre debates ativos e essenciais que ocorrem atualmente no âmbito da maior iniciativa universal de atenção primária à saúde comunitária do mundo.

Embora muitos aspectos da evolução e do impacto da ESF estejam bem documentados, um ponto abordado no ensaio me impressionou particularmente: apesar das pressões crescentes - sejam financeiras, políticas ou operacionais - a visão da ESF permaneceu profundamente enraizada no espírito humanitário e igualitário da *Alma Ata*. A posterior incorporação das equipes de saúde bucal, o reconhecimento da necessidade de maior multidisciplinaridade por meio dos Núcleos Ampliados de Saúde da Família (NASF), a ênfase renovada na melhoria da qualidade e as inovações desenvolvidas no âmbito do Programa Mais Médicos, entre muitas outras, apontam para uma visão evolutiva e incremental do que a atenção primária à saúde pode e deve ser para enfrentar os complexos desafios de saúde da atualidade.

Evidentemente, nem todas essas iniciativas foram igualmente bem-sucedidas. Algumas ações - como as mudanças de 2017 na Política Nacional de Atenção Básica (PNAB), que levaram ao desenvolvimento de equipes de atenção primária mais limitadas e em regime de tempo parcial; a concepção reducionista de “comunidade” promovida pelo programa Previne Brasil; e o contínuo subfinanciamento do Sistema Único de Saúde (SUS) - poderiam, cada uma por si só, ter representado o fim da ESF ^4^. 

O artigo ^3^ também nos lembra dos muitos desafios que estão por vir. Entre eles, estão o subfinanciamento persistente; a disponibilidade e a distribuição de recursos humanos; a realização de um cuidado integral e coordenado; as desigualdades estruturais que continuam a gerar acentuadas iniquidades em saúde; e a incerteza permanente quanto ao caráter público do SUS. Alguns desses problemas são específicos do Brasil, mas a maioria não é.

Apesar desses desafios, é essa visão coletiva e abrangente que, na minha opinião, sustentou a ESF ao longo de regimes políticos radicalmente distintos, grandes crises financeiras e até mesmo uma pandemia global. Neste 30º aniversário, façamos uma pausa para olhar para trás com orgulho e gratidão, antes de retomar o árduo trabalho de concretizar a visão ambiciosa e valiosa de um modelo forte e resiliente de atenção primária à saúde - modelo este que continua a inspirar pessoas ao redor do mundo e que, a cada dia, se aproxima mais do cumprimento de sua grande promessa.

## Data Availability

Não se aplica.
